# Effectiveness and safety of auricular acupuncture on adjuvant analgesia in patients with total knee arthroplasty: a randomized sham-controlled trial

**DOI:** 10.3389/fneur.2024.1275192

**Published:** 2024-02-16

**Authors:** Xingshuo Zhang, Hao Chen, Jingqiao Li, Xingang Liu, Xuesong Wang, Pingju Xue, Miao Lin, Jidong Li, Yanfen She

**Affiliations:** ^1^School of Acupuncture-Moxibustion and Tuina, Hebei University of Chinese Medicine, Shijiazhuang, China; ^2^West Medical Center in Shijiazhuang, Shijiazhuang, China; ^3^Hebei International Joint Research Center for Dominant Diseases in Chinese Medicine and Acupuncture, Shijiazhuang, China

**Keywords:** total knee arthroplasty, adjuvant analgesia, auricular acupuncture, randomized clinical trial, pain management

## Abstract

**Objective:**

This study aimed to evaluate the effectiveness and safety of auricular acupuncture (AA) on postoperative analgesia, the degree of postoperative nausea, and the effect of inflammation after total knee arthroplasty (TKA).

**Methods:**

This was a single-center, placebo-controlled, randomized clinical trial. In total, 96 patients were randomly divided into an AA group with an indwelling intradermal needle (*n* = 48) and a sham auricular acupuncture (SAA) group with a non-penetrating placebo needle (*n* = 48). Intra-spinal anesthesia was adopted in both groups during surgery, and an epidural analgesic pump was implanted after surgery for 48 h. The primary outcome was the post-surgery visual analog score (VAS) of resting and movement states (at 6, 12 h and 1, 2, 3, 5, and 7 days). The secondary outcomes included additional doses of analgesic injection during the treatment, C-reactive protein (CRP) levels, erythrocyte sedimentation rate (ESR), and white blood cell (WBC) count on the 1st, 3rd, and 7th day after the operation, nausea on the 1st, 2nd, and 3rd day after the operation, the Hospital for Special Surgery Knee Score (HSS) on the 2nd and 12th week after the operation, and adverse events.

**Results:**

The VAS in the AA group at 6 h, 12 h, 2, 3, and 5 days after surgery were lower than those of the SAA group (*p* < 0.05). Among the secondary outcomes, the total dose of additional analgesic injection after surgery in the AA group was lower than that in the SAA group (*p* < 0.05). The serum CRP on the 1st day after operation in the AA group was lower than that in the SAA group (*p* < 0.05). The degree of nausea on 2nd day after surgery in the AA group was lower than that in the SAA group (*p* < 0.05). There was no significant difference in other outcomes (*p* > 0.05).

**Conclusion:**

In this study, AA was shown to be an effective and safe complementary and alternative therapy for pain relief after TKA, which was able to reduce the total postoperative dose of additional painkillers, decrease serum CRP 1 day after surgery, and improve the degree of postoperative nausea.

**Clinical trial registration:**

www.chictr.org.cn, ChiCTR2100054403.

## Introduction

Knee osteoarthritis (KOA) is a chronic degenerative disease characterized by articular cartilage damage and involves the whole joint tissue (1). KOA is very common in elderly patients. Knee deformity and swelling can directly affect the function of the lower limbs, cause a series of complications, and seriously affect the quality of life of elderly patients (2).

Patients suffering from KOA are mainly treated by surgery and analgesic drugs, while total knee arthroplasty (TKA) is mostly used for patients with advanced KOA. However, TKA is often accompanied by varying degrees of persistent pain, which causes physiological and psychological adverse effects, reducing the efficacy of surgery to a certain extent (3, 4). The use of analgesics during and after surgery is associated with numerous side effects, including nausea and vomiting, respiratory depression (5), liver and kidney injury, depression (6), and adverse cardiovascular events (7). Therefore, it is particularly important to explore a method that can effectively control pain and reduce the use of analgesics.

Previous studies have found that acupuncture is primarily used to treat pain, and acupuncture has significant effects on pain relief, with high safety and few side effects (8, 9). A previous meta-analysis demonstrated that auricular point pressing, a traditional acupuncture therapy, combined with conventional analgesia can effectively relieve postoperative pain and reduce the consumption of different types of analgesic drugs (10). According to the theory of bioholography, the auricle forms a holographic reflex path from the homologically connected neurons in the brain and acts through the holographic connection of neurons in the brain (11). Therefore, abnormalities in various parts of the body cause corresponding changes in the ear through the holographic reflex path. Similarly, in the case of pain, the stimulation of ear points will also be transmitted to the corresponding organs of the body through the holographic reflex path to achieve the purpose of analgesia, while intervention away from the surgical incision site can avoid infection.

Two non-TKA randomized controlled trials (RCTs) (12, 13) have compared the use of indwelling intradermal needles in postoperative analgesia, with the results demonstrating that indwelling intradermal needles are effective in postoperative analgesia. The needles (14–17) used in previous postoperative analgesia studies were 0.2–0.22 mm in diameter and 1.2–1.5 mm in length, but the needle used in this study was 0.4 mm in diameter and 2.1 mm in length, which is larger in diameter and longer in the body than the conventional needles; thus, it is hoped that the needle will also provide better analgesia. However, there has been no previous RCT to evaluate the analgesic effect of an indwelling intradermal needle after TKA.

The underlying cause of postoperative pain is the stimulation of inflammatory factors. Serum C-reactive protein (CRP) level, erythrocyte sedimentation rate (ESR), and white blood cell (WBC) count are routine blood indicators used to assess postoperative infection as they represent inflammatory and anti-inflammatory factors in the serum of postoperative trauma (18). When an infection occurs after surgery, these indicators rise significantly. However, due to factors such as their short half-life, these indicators alone cannot be used as inflammation indicators to determine postoperative infection. Therefore, a combination of more than two indicators should be used to improve the accuracy of predicting postoperative infection after TKA (19). Additionally, numerous studies have demonstrated a positive correlation between CRP and postoperative visual analog score (VAS), suggesting that CRP may serve as an objective indicator for evaluating the postoperative analgesic effect of TKA (20–22).

Therefore, we designed a prospective sham-RCT to provide a clinical basis for the efficacy and safety of auricular indwelling intradermal needles as adjuvant analgesia to reduce the use of analgesic drugs.

## Materials and methods

### Study design and participants

This study was a single-center placebo-controlled RCT. The patients in the AA group received an indwelling intradermal needle and were covered by a disposable opaque circular Band-Aid, whereas the patients in the SAA group only received the Band-Aid to cover five auricular acupoints. All eligible participants provided written informed consent before the trial. This study strictly followed the Declaration of Helsinki and SPIRIT guidelines. The study was approved by the Ethics Committee of Shijiazhuang West Medical Center (No. JXXYYLL006) and registered in the Chinese Clinical Trial Registry (ChiCTR2100054403). Patients who underwent TKA in the Department of Joint Surgery of the center from November 2021 to October 2022 were recruited by conversation.

This trial was based on the Guidelines for the Diagnosis and Treatment of Osteoarthritis (2018 edition) (23) issued by the Orthopaedic Branch of the Chinese Medical Association as the criteria for the diagnosis of KOA, including (1) recurrent knee pain within the last month; (2) X-ray films (standing or weight-bearing position) showed narrowing of the joint space, sclerosis and/or cystic degeneration of the subchondral bone, and osteophyte formation at the joint edge; (3) age ≥ 50 years old; (4) morning stiffness time ≤ 30 min; and (5) a bone rub (feeling) during activity.

The inclusion criteria were as follows: patients diagnosed with KOA; conservative treatment was ineffective and met the surgical indications of TKA (24); patients undergoing primary TKA; patients and their families were fully informed, and informed consent was obtained; and patients with clear consciousness who possessed the capacity to communicate.

The exclusion criteria were as follows: strong resistance to acupuncture therapy or a history of seasickness; accompanied by pain in other parts of the body; serious medical diseases such as severe cardiovascular and cerebrovascular diseases or liver and kidney dysfunction; and dependent on or allergic to narcotic drugs.

### Interventions

The patients were anesthetized by spinal anesthesia during surgery and implanted with an epidural analgesia pump after surgery. The specific equation of the analgesia pump included sufentanil citrate injection (No. H20054171, Yichang Renfu Pharmaceutical Co., Ltd.), ketorolac tropanol injection (No. H20052634, Shandong New Times Pharmaceutical Co., Ltd.), and granisetron hydrochloride injection (No. H20093415, Hebei Yipin Pharmaceutical Co., Ltd.) in 96 mL of 0.9% sodium chloride solution (the specific content of the injections was determined according to the patient’s weight, age, sex, and other factors). The conventional drug administration rate was 2 mL/h, the patient-controlled dose was 0.5 mL/15 min, and the analgesia time after operation was 48 h. Celecoxib capsules (No. H20203297, Shiyao Group Ouyi Pharmaceutical Co., Ltd.) were taken orally for 7 days after surgery at a dosage of 200 mg/capsule twice a day, 1 capsule once after breakfast and dinner.

**Figure 1:**
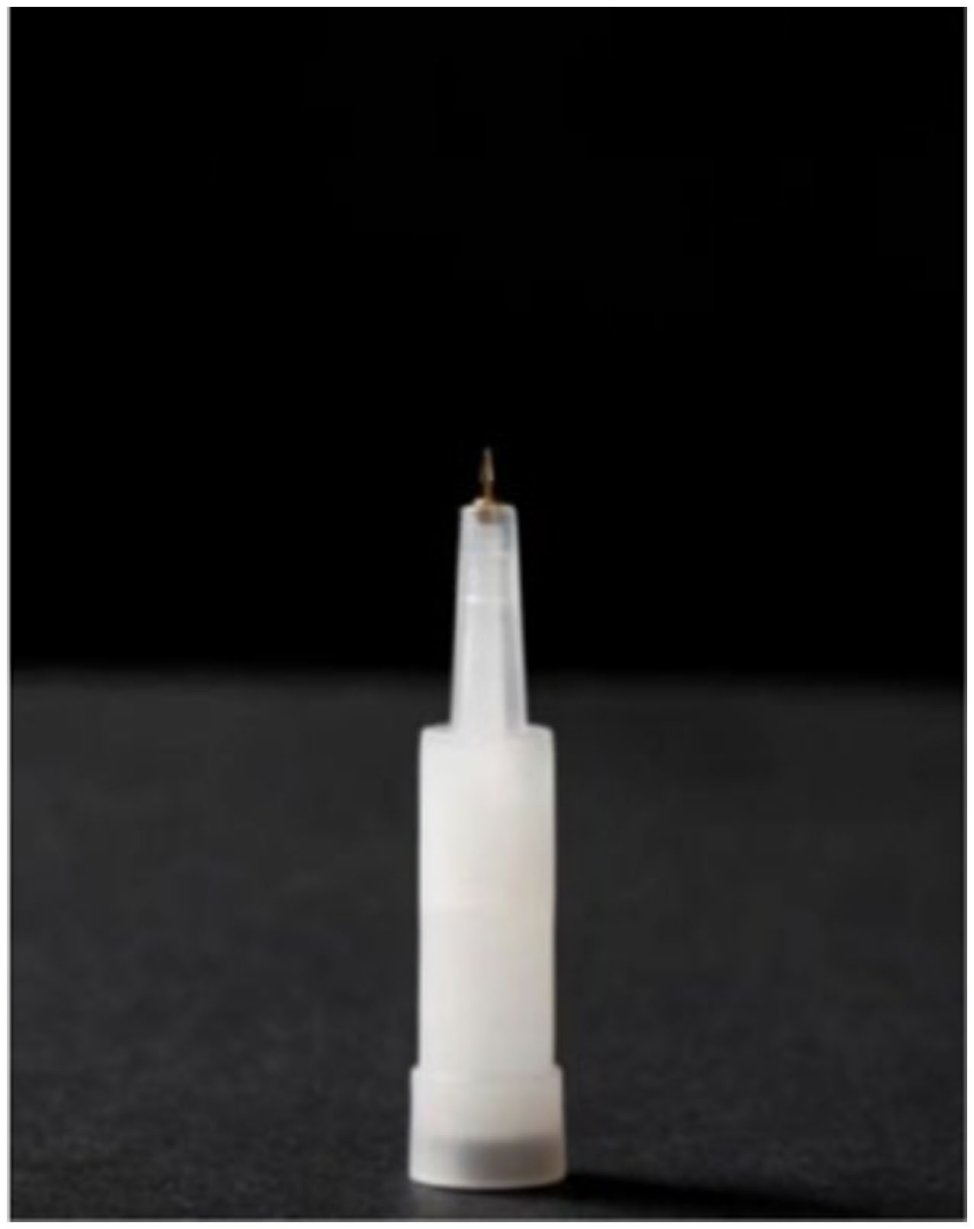
Indwelling intradermal needles.

**Figure 2:**
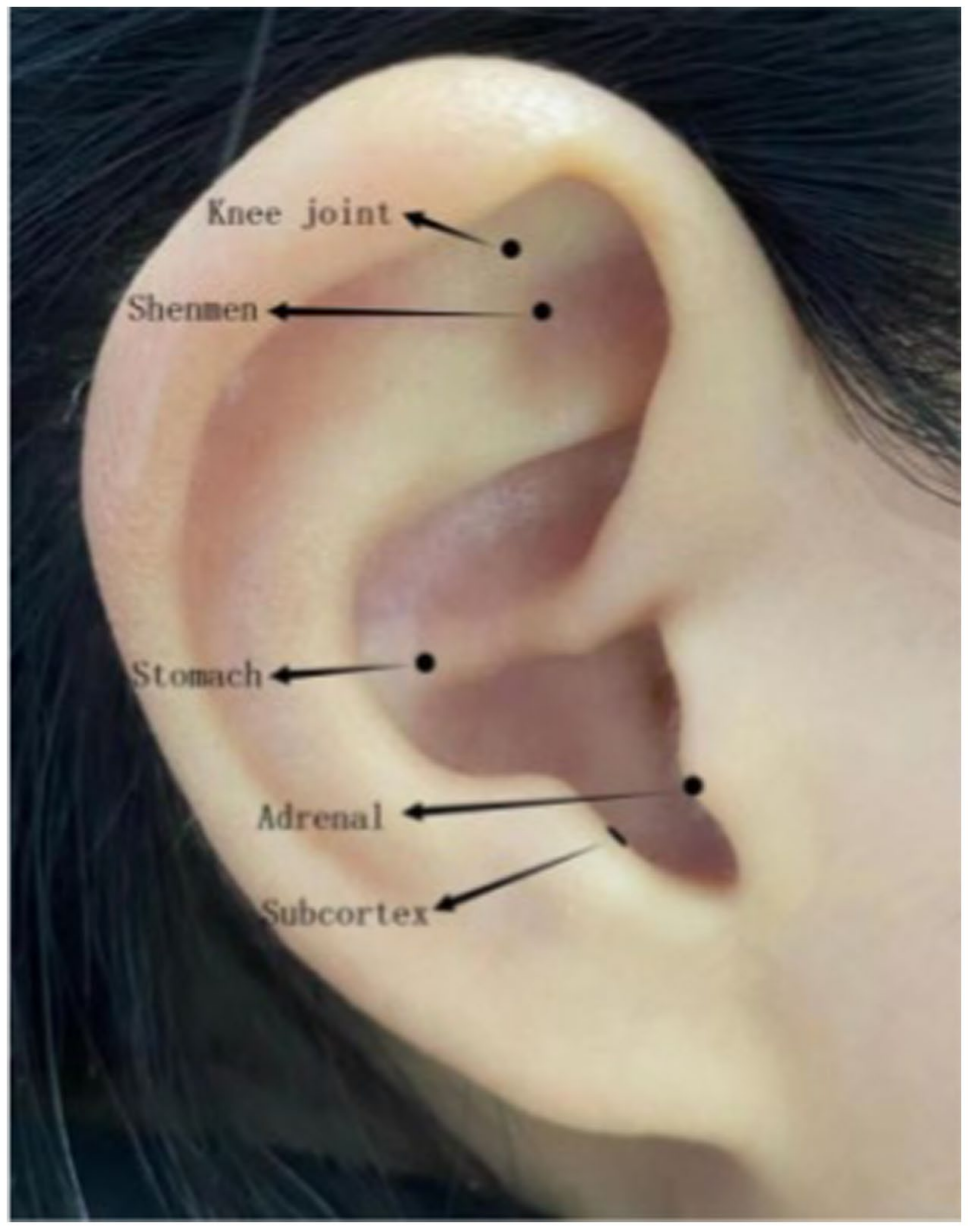
Selected auricular acupoints.

In the AA group, on the basis of the conventional treatments, the indwelling intradermal needles (type C, specification: 0.4 mm, 2.1 mm; Yushang Zhuzhun 20,202,200,203, Henan Kangjiulai Medical Technology Co., Ltd.; Figure 1) were inserted into five auricular acupoints, including those at the shenmen (TF_4_), adrenal gland (TG_2p_), knee (AH_4_), subcortex (AT_4_), and stomach (CO_4_) (25) (Figure 2, this right ear in the figure was provided by the first author). The needles were used on the operated side 1 day before the operation and on the contralateral side 3 days after the operation, lasting for 5 days. The treatment was performed under strict aseptic conditions by the same acupuncturist who had obtained the doctor’s certificate. After the needle was directly pricked into the selected auricular acupoints, a disposable opaque round Band-Aid was applied to cover the site. In the SAA group, a disposable opaque circular Band-Aid was applied to patch these acupoints (26, 27).

### Outcome measures

#### Primary outcome

The primary outcome was the VAS (28, 29) of resting and movement states at 6 h, 12 h, and 1, 2, 3, 5, and 7 days after surgery. The evaluation was conducted face to face by the evaluator and the patient using a VAS of 0–10 points. At the time of evaluation, the patient was shown a ruler with a scale of 10 cm, where 0 cm on one end indicated no pain, and 10 cm on the other end indicated the most pain. At each time point of the evaluation, the patient outlined the point that best represented the degree of pain, and this point was taken as the patient’s score.

#### Secondary outcomes

##### Additional analgesic injections

Parecoxib sodium for injection (No. H20193217, Hunan Cylon Pharmaceutical Co., Ltd., 40 mg/dose) was added when the pain score was greater than 6, and the total additional dose during the treatment period was recorded.

##### Hospital for special surgery knee score

The hospital for special surgery knee score (HSS) (30) was recorded when the patients were followed up by telephone or outpatient visits at 2 and 12 weeks postoperatively.

##### Serum examination

The CRP level, ESR, and WBC count in the two groups were detected at 1, 3, and 7 days after the operation. CRP was detected by immunoturbidimetry, ESR by Wei’s method, and WBC by semiconductor laser flow cytometry.

##### Degree of postoperative nausea

The VAS was used to record nausea at 1, 2, and 3 days after surgery (31, 32) when the evaluator was face to face with the patient. The VAS scale ranged from 0 to 10 points. At each time point of the evaluation, the point that best represented the degree of nausea was outlined by the patient, and this point was recorded as the patient’s score.

##### Safety assessments

The adverse reactions of the two groups during the treatment were observed and recorded; these included whether skin allergy, subcutaneous hematoma, local infection, and fainting occurred during acupuncture in the AA group and whether skin allergy occurred in the SAA group.

### Sample size calculation

Sample size calculations were determined from known literature (33, 34). The ratio between the two groups was 1:1 according to the following equation:


(1) $$n=\left[{\left(Z_{\alpha }+Z_{\beta }\right)}^{2}\times \left(1+1/k\right)\times P\left(1-P\right)\right]/{\left(P_{1}-P_{2}\right)}^{2}$$

where n is the required number of cases in each group, *P*_1_ and *P*_2_ represent the estimated values of the AA group and SAA group, respectively (represented by decimal points), and *P* is the combined rate. If the number of the two groups is equal (i.e., *k* = 1), *P* = (*P*_1_ + *P*_2_)/2. Type I error probability was set to α = 0.05, and type II error probability was set to β = 0.10; therefore, according to our clinical experience and the literature, Z_β_ = 1.645, Z_α_ = 1.282, *P*_1_ = 95.18%, and *P*_2_ = 70.22%. n ≈ 40 can be calculated using Equation 1. As the patient required two treatments and a follow-up after discharge, we considered a dropout rate of 20%. A total of 96 patients were needed, 48 patients in each group.

### Randomization, allocation concealment, and blinding

The enrolled patients were randomly assigned at a 1:1 ratio to the AA or SAA group using a random number table method generated by IBM SPSS 25.0 software, with treatments fixed in sequentially numbered opaque envelopes. When a patient was admitted for elective surgery, a physician who was not involved in the assessment opened the envelope to identify the patient group and informed the nurse to arrange the corresponding ward. The day before surgery, the patients were informed that they would receive auricular acupuncture at specific acupoints in addition to standard postoperative analgesia. After obtaining informed consent from the patient, the acupuncturist began the intervention. The acupuncturist had no further personal contact with the included patients at the end of each intervention. A professional orthopedics clinician, who had received training before the trial, was selected as the evaluator. The evaluators did not intervene and were unaware of the grouping of the patients. Similarly, the patients were also unaware of their grouping. Case collectors, acupuncturists, and data analysts were kept separate.

### Statistical analysis

Data were analyzed using IBM SPSS 25.0 software analysis. Measurement data were first analyzed to determine whether they followed a normal distribution using a Shapiro–Wilk normality test. If the data had a normal distribution, they were expressed as the mean ± standard deviation ( $\bar{x}$ ± s), and if they did not, then they were expressed as the median (upper quartile, lower quartile) [M (P25, P75)]. Comparisons between the two groups were performed using the Kruskal–Wallis test; intra-group comparisons were conducted using the Wilcoxon signed-rank test or Friedman test; and different time points were compared through repeated-measures analysis of variance between groups. Count data are described by the number of cases and percentage, and the chi-square test was used to compare the differences between the two groups. Two-sided *p*-values <0.05 were considered statistically significant.

## Results

### Baseline characteristics

**Figure 3:**
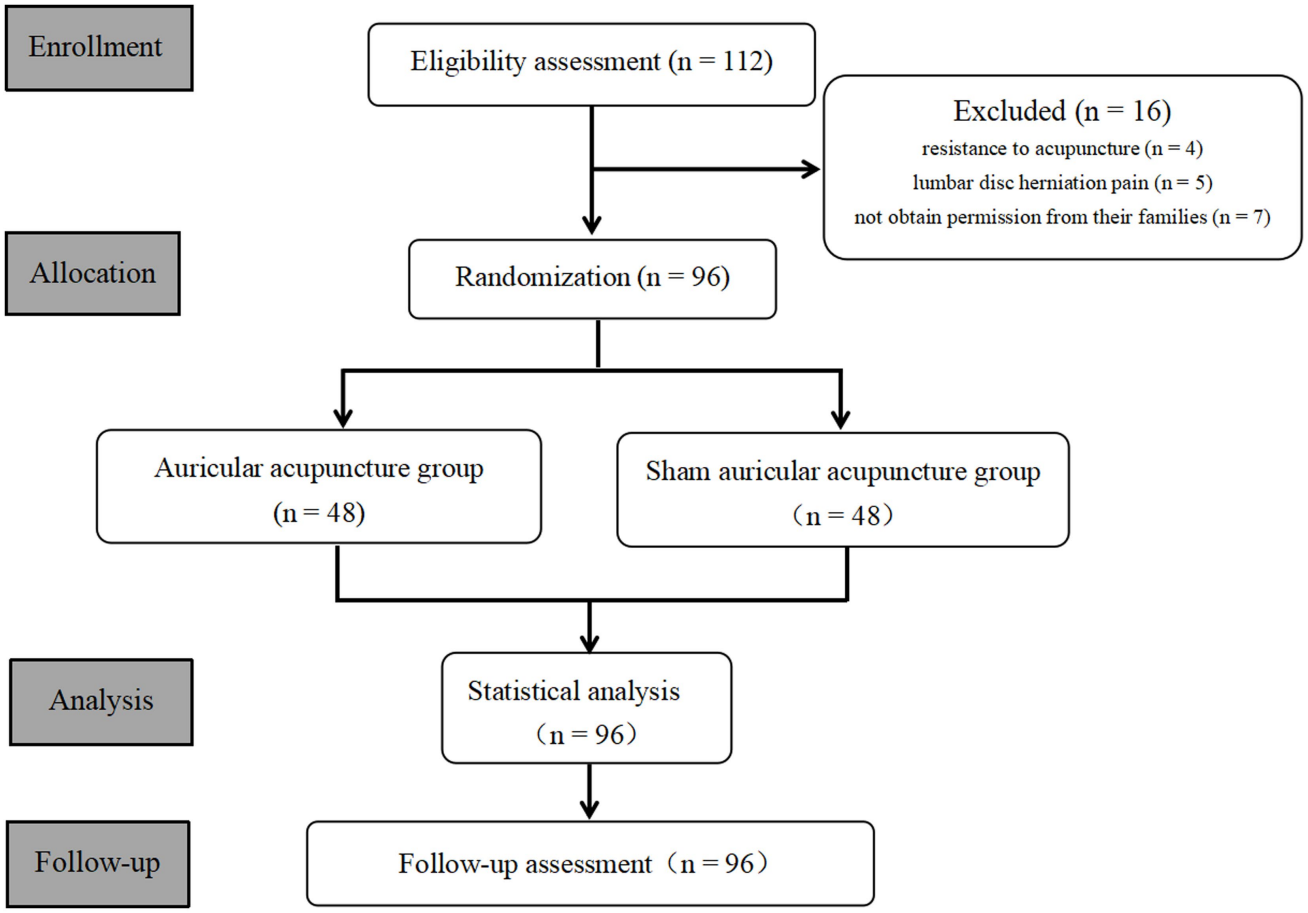
Flow chart of this trial.

A total of 112 patients were recruited, and 16 were excluded due to meeting the exclusion criteria. The flow chart of the study is shown in Figure 3. Because the patients were hospitalized during the treatment period and the later follow-up was in the form of re-consultation, there were no cases of dropout. The two groups were comparable, with no significant differences at baseline. All baseline demographic characteristics are shown in Table 1.

**Table 1:** Baseline characteristics of the population.

**Characteristics**	**Auricular acupuncture group** (*n* = 48)	**Sham auricular acupuncture group** (*n* = 48)
Age, year	67.9 ± 6.6	66.5 ± 7.5
Sex (male/female)	20/28	9/39
Weight, kg	71.69 ± 12.02	71.89 ± 12.12
Height, cm	163.02 ± 7.24	160.13 ± 5.54
Course of disease, year	10.5 (6.58, 15)	10.75 (7.25, 16)
Hypertension	19 (19.8%)	14 (14.6%)
Type 2 diabetes	4 (4%)	3 (3%)
Hypertension and type 2 diabetes	3 (3%)	2 (2%)
None	21 (21.9%)	29 (30.2%)
Initial pain, visual analog scale score	6.36 ± 1.77	6.50 ± 1.60
Left	29 (30%)	25 (26%)
Right	19 (20%)	23 (24%)
C-reactive protein, mg/L	1.82 (0.98, 2.66)	2.14 (0.88, 4.23)
Erythrocyte sedimentation rate, mm/h	17 (10, 26.75)	16.5 (11, 34.5)
White blood cell count, cell/μL	5.70 ± 1.65	5.95 ± 1.72

### Primary outcome

**Figure 4:**
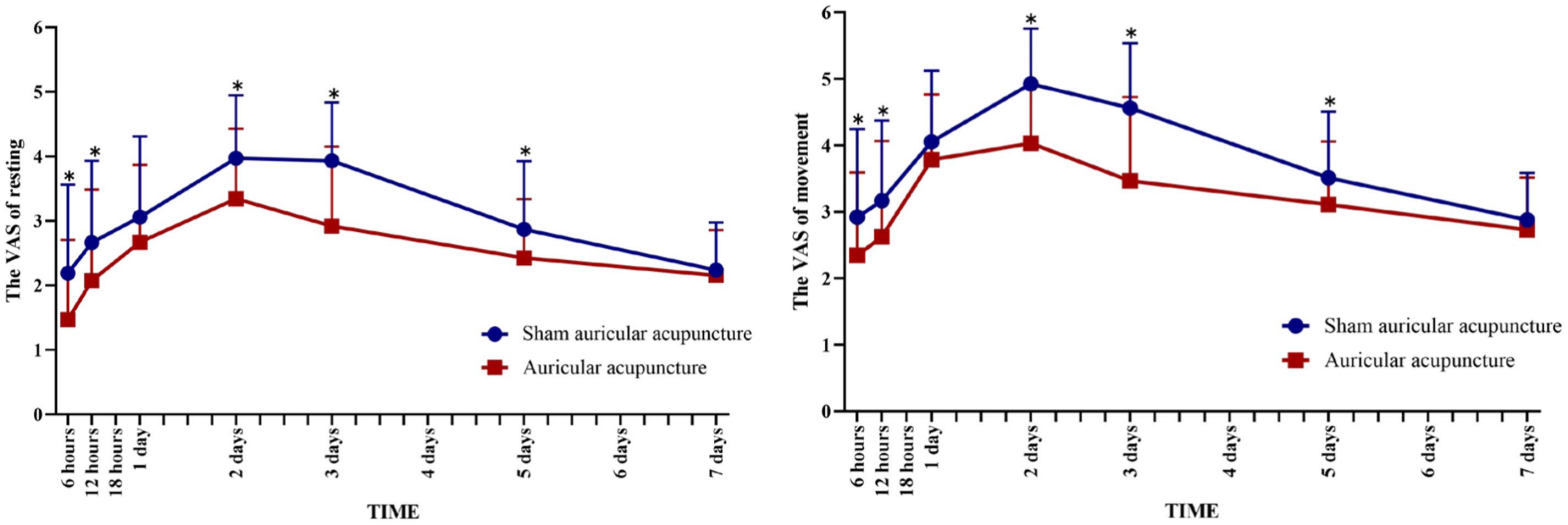
Visual analog scale scores over time in the AA and SAA groups. ^*^Compared with sham auricular acupuncture, ^*^P < 0.05.

The VAS values of the resting and movement states were significantly different between the two groups at 6 h, 12 h, 2 days, 3 days, and 5 days after the operation. The VAS values of patients who received AA were lower than those of the SAA group (*p* < 0.05, Figure 4), but there was no significant difference between the two groups at 1 day and 7 days after the operation (*p* > 0.05, Table 2).

**Table 2:** Comparison of the postoperative VAS values during resting and movement states at each time point between the two groups.

	**Auricular acupuncture group (*n* = 48)**	**Sham auricular acupuncture group (*n* = 48)**	***p-value***
Post-operative VAS value at resting state, score			
6 h	1.47 ± 1.24	2.19 ± 1.37	0.008
12 h	2.08 ± 1.41	2.67 ± 1.27	0.034
1 day	2.68 ± 1.20	3.06 ± 1.26	0.125
2 days	3.34 ± 1.09	3.98 ± 0.98	0.004
3 days	2.92 ± 1.23	3.94 ± 0.90	0.000
5 days	2.43 ± 0.91	2.87 ± 1.06	0.030
7 days	2.16 ± 0.70	2.24 ± 0.74	0.581
Post-operative VAS value at movement state, score			
6 h	2.35 ± 1.24	2.92 ± 1.32	0.031
12 h	2.63 ± 1.44	3.17 ± 1.20	0.048
1 day	3.79 ± 0.98	4.05 ± 1.07	0.203
2 days	4.03 ± 0.90	4.93 ± 0.83	0.000
3 days	3.47 ± 1.26	4.56 ± 0.98	0.000
5 days	3.11 ± 0.95	3.51 ± 0.99	0.045
7 days	2.73 ± 0.78	2.89 ± 0.70	0.326

### Secondary outcomes

The total dosage of analgesic requirement after the operation in the AA group was lower than that in the SAA group (*p* < 0.05), and the additional dosage at each time point in the AA group was also lower than that in the SAA group postoperative days 1 and 2 (*p* < 0.05) as shown in Table 3.

**Table 3:** Comparison of the postoperative dose of additional analgesic injections between the two groups.

Additional dosage at each time point	Auricular acupuncture group (*n* = 48)	Sham auricular acupuncture group (*n* = 48)	***P-value***
6 h	1 (0, 1)	1 (1, 1)	0.348
12 h	0 (0, 1)	0 (0, 1)	0.943
1 day	0 (0, 1)	0 (0, 1)	0.044
2 days	0 (0, 0)	0 (0, 1)	0.019
3 days	0 (0, 0)	0 (0, 0)	0.103
5 days	0 (0, 0)	0 (0, 0)	0.313
7 days	0 (0, 0)	0 (0, 0)	0.058
Total additional injections	3 (1, 4)	1 (1, 2.5)	0.004

The level of CRP in the AA group was lower than that in the SAA group on the 1st day after the operation (*P* < 0.05), while there was no significant difference in CRP between the two groups on the 3rd and 7th days after the operation (*P* > 0.05). In addition, there was no significant difference in ESR and WBC between the two groups at 1, 3, and 7 days after the operation (*p* > 0.05, Table 4).

**Table 4:** Comparison of the postoperative serum CRP, ESR, and WBC between the two groups.

C-reactive protein	Auricular acupuncture group (*n* = 48)	Sham auricular acupuncture group (*n* = 48)	***P-value***
1 day	67.25 ± 32.20	80.79 ± 34.43	0.049
3 days	147.53 ± 52.95	155.36 ± 52.95	0.446
7 days	64.39 ± 33.18	62.08 ± 28.38	0.715
Erythrocyte sedimentation rate			
1 day	25 (15, 35.75)	20 (15.25, 30)	0.359
3 days	93.50 ± 29.28	92.56 ± 24.07	0.864
7 days	93.38 ± 26.53	99.29 ± 30.60	0.314
White blood cell count			
1 day	11.47 ± 3.33	11.98 ± 3.38	0.459
3 days	9.30 ± 2.73	9.34 ± 2.30	0.955
7 days	7.38 ± 1.87	7.74 ± 2.17	0.381

The degree of nausea in the AA group was lower than that in the SAA group on the 2nd day after the operation (*p* < 0.05), but there was no significant difference on the 1st and 3rd days after the operation (*p* > 0.05, Table 5).

**Table 5:** Comparison of postoperative nausea between the two groups.

Degree of postoperative nausea	Auricular acupuncture group (*n* = 48)	Sham auricular acupuncture group (*n* = 48)	***P-value***
1 day	1 (0, 2.5)	2.3 (0, 4.08)	0.062
2 days	1.25 (0, 2.3)	1.7 (0.08, 3.38)	0.046
3 days	0 (0, 1)	1 (0, 1.73)	0.081

The HSS was not significantly different between the two groups (*p* > 0.05, Table 6).

**Table 6:** Follow-up results of HSS scores in the two groups.

Postoperative HSS score	Auricular acupuncture group (*n* = 48)	Sham auricular acupuncture group (*n* = 48)	***P-value***
2 weeks	95.5 (88, 100)	98 (90, 100)	0.348
12 weeks	100 (100, 100)	100 (100, 100)	0.095

### Adverse events

During the treatment, none of the patients suffered any adverse events (e.g., infection). The safety was not significantly different between the two groups (*p* > 0.05).

## Discussion

Approximately 10–34% of patients with TKA have poor acute pain control, leading to chronic pain (35), which seriously affects the postoperative rehabilitation and quality of life of the patients and increases their reliance on analgesics. However, the drug-related side effects of analgesics limit their application in daily clinical practice (36). Therefore, it is particularly important to explore an analgesic method with the characteristics of green therapy (37).

As a kind of green complementary and alternative therapy, auricular acupuncture therapy has a good analgesic effect during the perioperative period and has been widely used in gynecology, orthopedics, anorectal, and other fields (10). According to the theory of bioholography, the close association between auricular acupoints and zangfu-meridians is the basis for the clinical treatment of pain (11).

The pain mechanism of TKA can be attributed to peripheral sensitization and central sensitization (38). Peripheral sensitization is caused by injury to the body, resulting in inflammation and local organ pain. On the other hand, central sensitization occurs due to pain stimulation, leading to increased neuronal excitability in the dorsal horn of the spinal cord and resulting in pain. Research has demonstrated that the application of auricular acupuncture can activate the descending pain inhibition pathway along the dorsal side of the spinal cord, specifically where the dorsal horn cells are located. This activation plays an analgesic role and effectively relieves pain after TKA (39). In addition, the analgesic effect of auricular acupuncture also works by stimulating specific acupoints on the ear to activate the nociceptin receptors, which release endogenous morphine-like substances such as endorphins and enkephalins, thereby enhancing the pain threshold and reducing the degree of pain (40). Our results demonstrated that an indwelling intradermal needle, as an auricular acupuncture device, could provide effective postoperative analgesia after TKA. The best analgesic effect was achieved 2 days after the operation, which reduced moderate pain (VAS score 4–6 points) to mild pain (VAS score 0–3 points), as well as the dose of analgesic needling.

Based on previous literature, textbooks, and clinical practice experience of acupuncture, the auricular acupoints stimulated in this study included the TF_4_, TG_2p_, AH_4_, AT_4_, and CO_4_. First, according to the results of literature data mining (41, 42), TF_4_ and AT_4_ were the two most commonly used in perioperative analgesia. The TG_2p_ can effectively regulate the patient’s autonomic nerve with sedative and analgesic effects (43). Second, after the operation, the vital qi of the body is consumed and the qi of the spleen and stomach is gradually weakened, causing the stomach qi to be out of balance. At the same time, the usage of analgesics stimulates the stomach organ, which leads to nausea and vomiting. The CO_4_ was selected to control nausea and vomiting based on clinical experience. Third, according to the location of the lesion, the AH_4_ was directly stimulated to relieve the pain, which is a type of acupoint selection method based on traditional Chinese medicine. All in all, the combination of five acupoints can regulate the meridians and collateralis to reduce pain.

It is found that CRP, ESR, and WBC are the primary inflammatory and anti-inflammatory factors in the serum of patients who have suffered postoperative trauma. Those blood indicators may be an objective index for the evaluation of postoperative analgesia after TKA. Our results showed that AA could reduce the CRP level over a certain period of time, which showed that the analgesic effect of intradermal needling may be the result of an anti-inflammation effect; however, ESR and WBC did not decrease significantly. The reason for this could be that the diagnostic advantage of CRP is significantly higher than that of the ESR and WBC count (44). The ESR and WBC are often used as monitoring indices to evaluate postoperative infection; however, as the specificity of these indices is relatively low and easily affected by many factors (45), this result still needs to be verified by a larger sample size.

The degree of postoperative nausea in the AA group was lower than that in the SAA group at 2 days after the operation, partly because fewer analgesic needles were added in the AA group, especially at 2 days after the operation. According to the perspective of Chinese medicine, the pathogenesis of vomiting is a “stomach disorder causing qi inverse to the upper.” Patients with TKA have a deficiency of both qi and blood of the body, which impairs the function of the spleen and stomach qi and the occurrence and aggravation of nausea induced by qi inverse (46). In addition, the risk of nausea is increased due to the aggravation of postoperative pain in patients. Simultaneously, the use of analgesics stimulates the vomiting center of the brain, which will also induce the generation and aggravation of nausea (47). Modern research has proved that auricular acupoint therapy can effectively alleviate the degree of postoperative nausea (47). Moreover, it has been verified that CO_4_, one of the five acupoints selected in this study, can reduce regurgitation and stop vomiting.

### Limitations

First, the population of this trial was taken from a single center, which may cause population bias. In future, our research team will conduct a multicenter RCT with a large sample size. Second, this pilot study only examined the visual analog score as the primary outcome measure of postoperative pain but did not consider the possible influencing factors such as perioperative patients’ emotional state and cognitive function on pain assessment. The effect of these factors on the results is unknown. Third, this study was invasive and may not have been double-blind. Despite the implementation of the principle of three separations, the effect of blind implementation was not assessed, which is an important point for future consideration of sham control settings. Fourth, the study should be conducted in experienced institutions to obtain more rigorous data. Fifth, the research team will consider how to assess the analgesia-related effects of indwelling intradermal needles independently of the use of analgesics. In parallel, we will seek to provide more reliable and effective clinical methods for postoperative analgesia in TKA based on this study in the future.

## Conclusion

Auricular acupuncture is effective for adjuvant analgesia after TKA and functions by reducing CRP on the first day after the operation, the use of analgesic needles, and the occurrence of postoperative nausea.

## Data availability statement

The raw data supporting the conclusions of this article will be made available by the authors, without undue reservation.

## Ethics statement

The studies involving humans were approved by Ethics Committee of Shijiazhuang West Medical Center. The studies were conducted in accordance with the local legislation and institutional requirements. The participants provided their written informed consent to participate in this study. Written informed consent was obtained from the individual(s) for the publication of any potentially identifiable images or data included in this article.

## Author contributions

XZ: Writing – original draft. HC: Writing – original draft. JQL: Data curation, Visualization, Writing – review & editing. XL: Funding acquisition, Resources, Writing – review & editing. PX: Conceptualization, Methodology, Writing – review & editing. ML: Formal analysis, Writing – review & editing. JDL: Funding acquisition, Resources, Supervision, Writing – review & editing. YS: Conceptualization, Funding acquisition, Methodology, Resources, Visualization, Writing – review & editing. XW: Methodology, Software.
